# Supplementary material for: Evolutionary game theory modelling to represent the behavioural dynamics of economic shutdowns and shield immunity in the COVID-19 pandemic
Source: R Soc Open Sci. 2020 Sep 30;7(9):201095. doi: 10.1098/rsos.201095 (PMC7540740; doi:10.1098/rsos.201095)
Supplement: Code [file rsos201095supp1.pdf]

```cpp
// COVID 19 cost.cpp : K M ARIFUL KABIR 4th order RK method

#include "stdafx.h"
#include <iostream>
#include <fstream>
#include <stdio.h>
#include <math.h>
#include <vector>
#include <list>
#include <string.h>
#include <stdlib.h>
#include <ctype.h>
#include <sstream>
#include <string>
#include <cstdio>
#include <time.h>
using namespace std;

int main()
{
# define times 500

# define dt 0.001

# define B 2.0// transmission rate

# define alpha 1./6.// Incubation rate to be infective

# define Gs 1./10.// Recovery rate (from symptomatic)

# define Ga 1./6.//Recovery rate (from asymptomatic)

# define Gh 1./18.//Recovery rate (from hospital)

# define r 0.1//testing rate/ hospitalized rate

# define delta 1./30.//Quarantine to exposed rate

# define theta 0.0//Shield -immunity factor

# define h 1.0//Hospital facilities factor

# define rho 0.5//Asymptomatic infection rate

# define Cd 1.0//Relative cost of lockdown

# define m 1.0// rate
```

```
# define q0 1.0//rate

        ostringstream file1;

        file1<<"COVID 19 model"<<".csv";

        ofstream Data1(file1.str().c_str(),ios_base::out|ios_base::trunc);

        Data1<<"t, S, Tot I, Q, H, E, R"<<endl;

        vector<double>S(70000, 0);

        vector<double>E(70000, 0);

        vector<double>Q(70000, 0);

        vector<double>IS(70000, 0);

        vector<double>IA(70000, 0);

        vector<double>H(70000, 0);

        vector<double>R(70000, 0);

        vector<double>X(70000, 0);

        vector<double>QQ(70000, 0);

        S[0] = 1.0-0.0002;
        E[0] = 0.00;
        Q[0] = 0.0;
        IS[0] = 0.0001;
        IA[0] = 0.0001;
        H[0] = 0.0;
        R[0] = 0.0;
        X[0] = 0.0001;

        double i = 0.;

        while (i < times) {

                double eta = X[i];

                QQ[i] = Cd * q0*(1 - exp(-i));
                double q = QQ[i];
```

```
		double	KS1 = -B * ((S[i] * (IA[i] + IS[i] + q * Q[i] + h * H[i])) / (1 + theta * R[i]));
		double	KE1 = B * ((S[i] * (IA[i] + IS[i] + q * Q[i] + h * H[i])) / (1 + theta * R[i])) - alpha * E[i] + delta * Q[i];
		double	KQ1 = alpha * eta *E[i] - delta * Q[i];
		double	KIS1 = alpha * (1 - eta)*(1 - rho) *E[i] - Gs * IS[i] - r * IS[i];
		double	KIA1 = alpha * (1 - eta)*rho *E[i] - Ga * IA[i];
		double	KH1 = r * IS[i] - Gh * H[i];
		double	KR1 = Gs * IS[i] + Ga * IA[i] + Gh * H[i];

		double	KX1 = m * X[i] * (1 - X[i])*(-Cd * Q[i] + IS[i] + IA[i]);


		double	KS2 = -B * (S[i] + KS1 * dt*0.5) * ((IA[i] + KIA1 * dt*0.5) + (IS[i] + KIS1 * dt*0.5) + q * (Q[i] + KQ1 * dt*0.5) + h * (H[i] + KH1 * dt*0.5)) / (1 + theta * (R[i] + KR1 * dt*0.5));
		double	KE2 = B * ((S[i] + KS1 * dt*0.5) * ((IA[i] + KIA1 * dt*0.5) + (IS[i] + KIS1 * dt*0.5) + q * (Q[i] + KQ1 * dt*0.5) + h * (H[i] + KH1 * dt*0.5)) / (1 + theta * (R[i] + KR1 * dt*0.5))) - alpha * (E[i] + KE1 * dt*0.5) + delta * (Q[i] + KQ1 * dt*0.5);
		double	KQ2 = alpha * eta *(E[i] + KE1 * dt*0.5) - delta * (Q[i] + KQ1 * dt*0.5);
		double	KIS2 = alpha * (1 - eta)*(1 - rho) *(E[i] + KE1 * dt*0.5) - Gs * (IS[i] + KIS1 * dt*0.5) - r * (IS[i] + KIS1 * dt*0.5);
		double	KIA2 = alpha * (1 - eta)*rho *(E[i] + KE1 * dt*0.5) - Ga * (IA[i] + KIA1 * dt*0.5);
		double	KH2 = r * (IS[i] + KIS1 * dt*0.5) - Gh * (H[i] + KH1 * dt*0.5);
		double	KR2 = Gs * (IS[i] + KIS1 * dt*0.5) + Ga * (IA[i] + KIA1 * dt*0.5) + Gh * (H[i] + KH1 * dt*0.5);

		double	KX2 = m * (X[i] + KX1 * dt*0.5) * (1 - (X[i] + KX1 * dt*0.5))*(-Cd * (Q[i] + KQ1 * dt*0.5) + (IS[i] + KIS1 * dt*0.5) + (IA[i] + KIA1 * dt*0.5));


		double	KS3 = -B * (S[i] + KS2 * dt*0.5) * ((IA[i] + KIA2 * dt*0.5) + (IS[i] + KIS2 * dt*0.5) + q * (Q[i] + KQ2 * dt*0.5) + h * (H[i] + KH2 * dt*0.5)) / (1 + theta * (R[i] + KR2 * dt*0.5));
		double	KE3 = B * ((S[i] + KS2 * dt*0.5) * ((IA[i] + KIA2 * dt*0.5) + (IS[i] + KIS2 * dt*0.5) + q * (Q[i] + KQ2 * dt*0.5) + h * (H[i] + KH2 * dt*0.5)) / (1 + theta * (R[i] + KR2 * dt*0.5))) - alpha * (E[i] + KE2 * dt*0.5) + delta * (Q[i] + KQ2 * dt*0.5);
```

```
            double      KQ3 = alpha * eta * (E[i] + KE2 * dt*0.5) - delta *
(Q[i] + KQ2 * dt*0.5);
            double      KIS3 = alpha * (1 - eta)*(1 - rho) *(E[i] + KE2 *
dt*0.5) - Gs * (IS[i] + KIS2 * dt*0.5) - r * (IS[i] + KIS2 * dt*0.5);
            double      KIA3 = alpha * (1 - eta)*rho *(E[i] + KE2 * dt*0.5) - Ga
* (IA[i] + KIA2 * dt*0.5);
            double      KH3 = r * (IS[i] + KIS2 * dt*0.5) - Gh * (H[i] + KH2 *
dt*0.5);
            double      KR3 = Gs * (IS[i] + KIS2 * dt*0.5) + Ga * (IA[i] + KIA2
* dt*0.5) + Gh * (H[i] + KH2 * dt*0.5);

            double      KX3 = m * (X[i] + KX2 * dt*0.5) * (1 - (X[i] + KX2 *
dt*0.5))*(-Cd * (Q[i] + KQ2 * dt*0.5) + (IS[i] + KIS2 * dt*0.5) + (IA[i] + KIA2
* dt*0.5));


            double      KS4 = -B * (S[i] + KS3 * dt) * ((IA[i] + KIA3 * dt) +
(IS[i] + KIS3 * dt) + q * (Q[i] + KQ3 * dt) + h * (H[i] + KH3 * dt)) / (1 +
theta * (R[i] + KR3 * dt));
        double    KE4 = B * ((S[i] + KS3 * dt) * ((IA[i] + KIA3 * dt) + (IS[i] +
KIS3 * dt) + q * (Q[i] + KQ3 * dt) + h * (H[i] + KH3 * dt)) / (1 + theta * (R[i]
+ KR3 * dt))) - alpha * (E[i] + KE3 * dt) + delta * (Q[i] + KQ3 * dt);
        double    KQ4 =  alpha * eta *(E[i] + KE3 * dt) - delta * (Q[i] + KQ3 *
dt);
            double      KIS4 = alpha * (1 - eta)*(1 - rho) *(E[i] + KE3 * dt) -
Gs * (IS[i] + KIS3 * dt) - r * (IS[i] + KIS3 * dt);
            double      KIA4 = alpha * (1 - eta)* rho *(E[i] + KE3 * dt) - Ga *
(IA[i] + KIA3 * dt);
            double      KH4 = r * (IS[i] + KIS3 * dt) - Gh * (H[i] + KH3 * dt);
            double      KR4 = Gs * (IS[i] + KIS3 * dt) + Ga * (IA[i] + KIA3 *
dt) + Gh * (H[i] + KH3 * dt);

            double      KX4 = m * (X[i] + KX3 * dt) * (1 - (X[i] + KX3 * dt))*(-
Cd * (Q[i] + KQ3 * dt) + (IS[i] + KIS3 * dt) + (IA[i] + KIA3 * dt));


            S[i + dt] = S[i] + (dt / 6.0)*(KS1 + 2 * KS2 + 2 * KS3 + KS4);

            E[i + dt] = E[i] + (dt / 6.0)*(KE1 + 2 * KE2 + 2 * KE3 + KE4);

            Q[i + dt] = Q[i] + (dt / 6.0)*(KQ1 + 2 * KQ2 + 2 * KQ3 + KQ4);

            IS[i + dt] = IS[i] + (dt / 6.0)*(KIS1 + 2 * KIS2 + 2 * KIS3 + KIS4);

            IA[i + dt] = IA[i] + (dt / 6.0)*(KIA1 + 2 * KIA2 + 2 * KIA3 + KIA4);

            H[i + dt] = H[i] + (dt / 6.0)*(KH1 + 2 * KH2 + 2 * KH3 + KH4);
```

```
            R[i + dt] = R[i] + (dt / 6.0)*(KR1 + 2 * KR2 + 2 * KR3 + KR4);

            X[i + dt] = X[i] + (dt / 6.0)*(KX1 + 2 * KX2 + 2 * KX3 + KX4);

                      Data1 << i << "," << S[i]<< ", " << IS[i] + IA[i] << ", "
<< Q[i] << "," << H[i] << ", " << E[i] << ", " << R[i] << endl;

                       cout << i << "," << S[i] << ", " << IS[i] + IA[i] << ",
" << Q[i] << "," << H[i] << ", " << E[i] << ", " << R[i] << endl;

                       i = i + dt;

                       }

      Data1.close();
}
```
